# Harlequin sign associated with Horner syndrome secondary to an 11 cm left upper lung lobe adenocarcinoma

**DOI:** 10.1016/j.jdcr.2023.10.017

**Published:** 2023-11-03

**Authors:** Yingjoy Li, Deborah J. Moon, Kenneth G. Linden

**Affiliations:** Department of Dermatology, University of California Irvine, Orange, California

**Keywords:** flushing, Harlequin sign, Horner syndrome, imaging, lung adenocarcinoma, malignancy, mediastinal

## Introduction

Harlequin sign presents as unilateral flushing and sweating of the face and may occur with Horner syndrome, a condition that involves cervical sympathetic and ocular dysfunction on the nonflushed side.^1^ The neuronal deficits resulting in Harlequin sign can be explained by a wide range of etiologies, making diagnosis a challenge. We present a rare case of Harlequin sign concomitant with Horner syndrome associated with underlying malignancy.

## Case

An 81-year-old female with newly diagnosed metastatic lung adenocarcinoma was transferred for palliative radiation therapy for a large left upper lung lobe adenocarcinoma and was noted to have right-sided facial flushing. Dermatology was consulted for further evaluation. Her review of systems was negative for pruritus, pain, tingling, burning, or blistering of the skin, arguing against zoster. She denied any neuropathic symptoms, changes in vision, flushing when eating, or prior procedures to the right side of her face that might otherwise suggest auriculotemporal syndrome.

**Fig 1:**
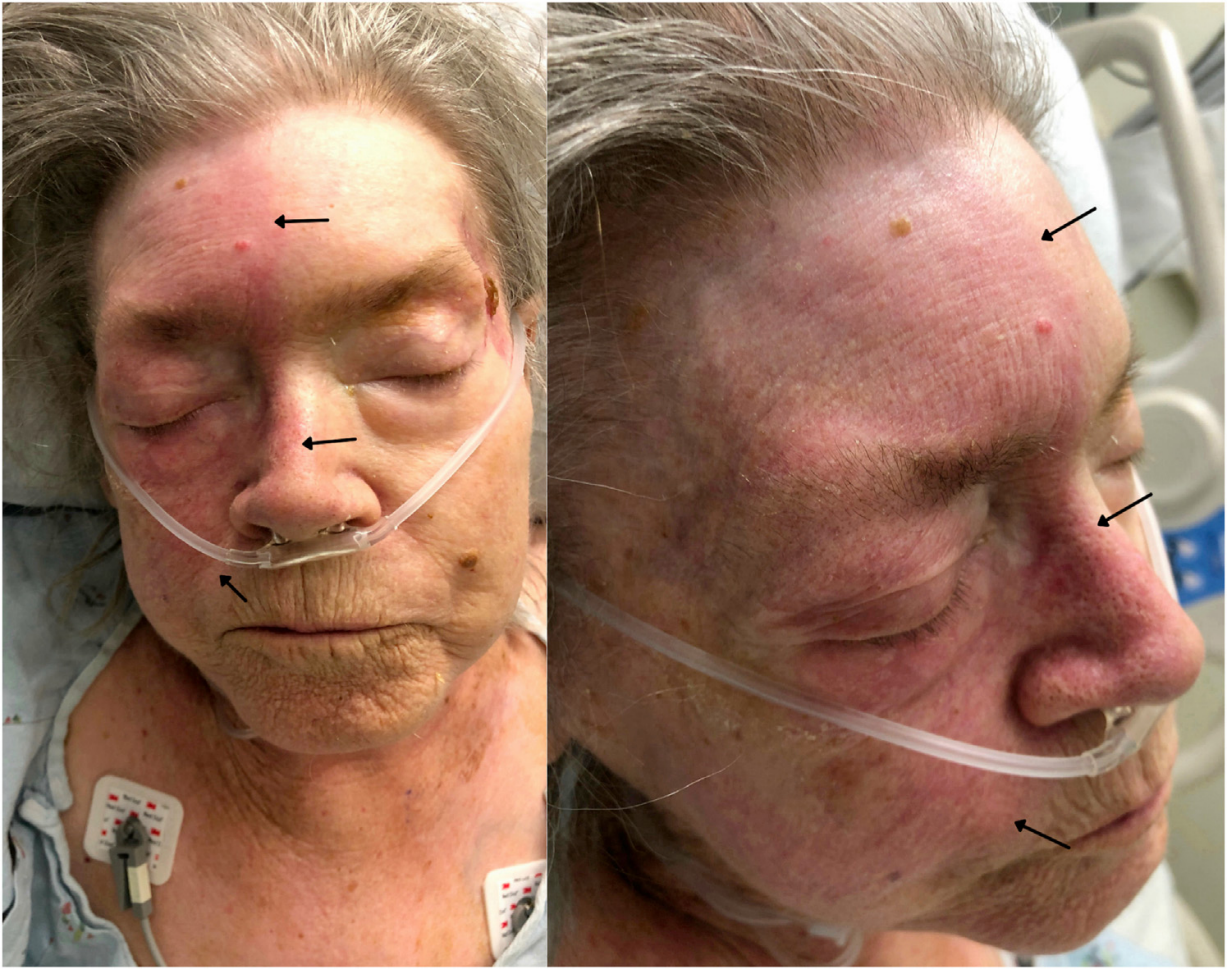
Hemifacial erythema distributed on the V1 and V2 dermatomes, sharply demarcated at the midline on the forehead and along the lower third of the face near the right nasolabial fold, indicated by the *black arrows*.

**Fig 2:**
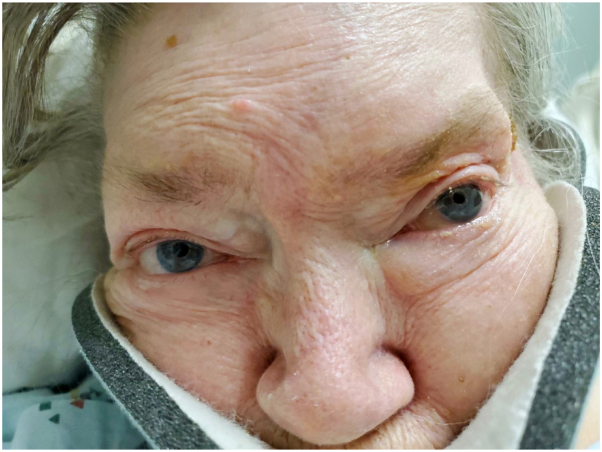
Partial ptosis of left upper eyelid and miosis of left pupil.

**Fig 3:**
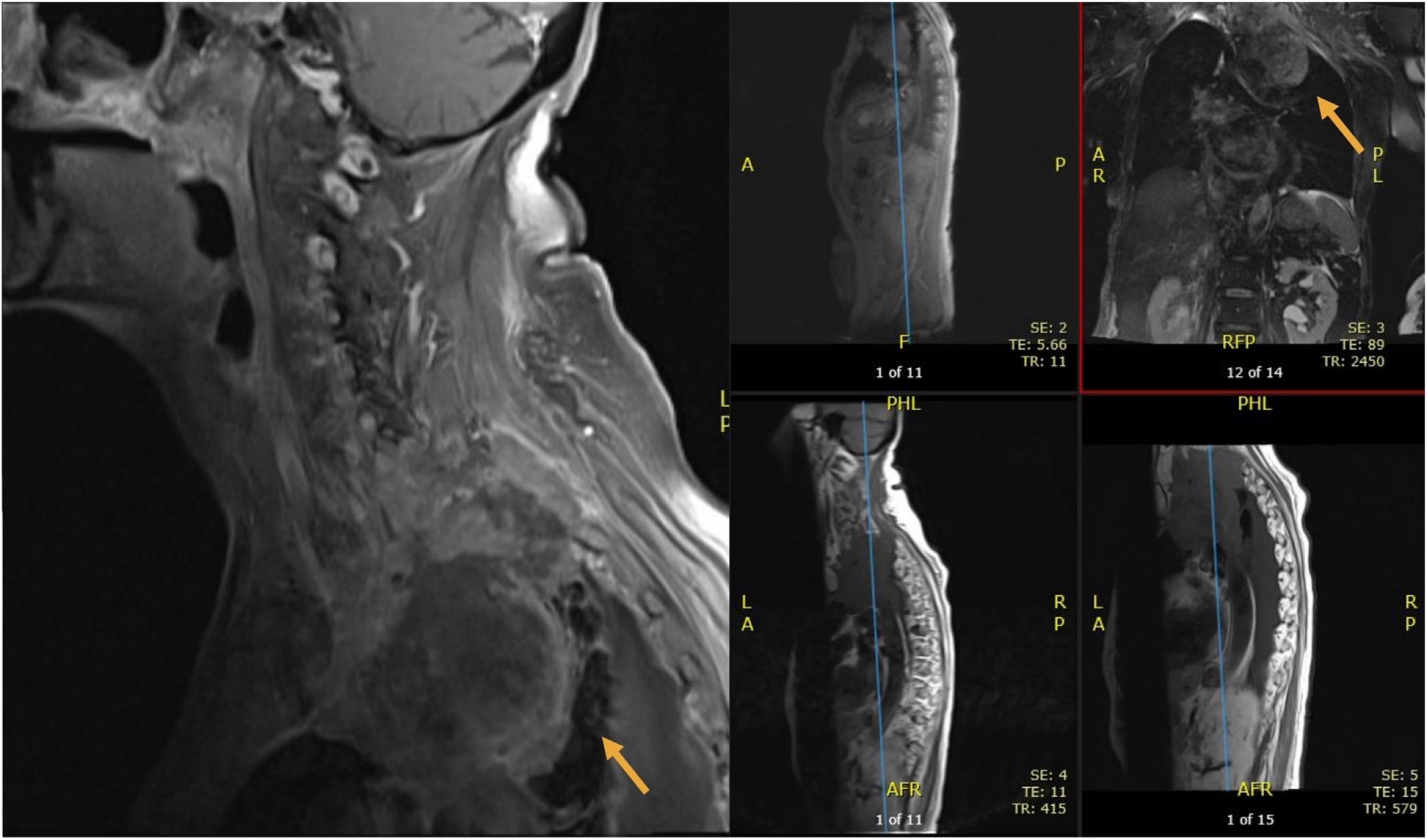
Thoracic MRI demonstrating a left upper lung lobe mass measuring 11.0 cm with encasement of the left common carotid artery and posterior elements of T1 and T2 segments. *MRI*, Magnetic resonance imaging.

Examination revealed unilateral facial erythema on the right side, predominantly in the V1 and V2 dermatomal distribution sharply demarcated at the midline (Fig 1). To rule out various dysautonomic conditions, a complete neurologic examination was performed. The patient had left pupillary miosis with partial ptosis that had started 4 months ago, although no asymmetric anhidrosis (Fig 2). Both pupils were round and reactive to light. There was clear atrophy, weakness, and decreased sensation in the upper left extremity compared with the right. The biceps, brachialis, triceps, and patellar reflexes were 2+ bilaterally. A cervical magnetic resonance imaging showed a large left apical pulmonary mass measuring 11.0 cm with encasement of the left common carotid artery and left subclavian artery, accompanied by invasion of the adjacent soft tissues of the neck, chest, and mediastinum. The mass also invaded through the vertebral body and posterior elements of T1 and T2 with partial encasement and mass effect of the spinal cord at these levels (Fig 3).

Given the constellation of clinical findings in the setting of a large left upper lung lobe tumor, the patient was diagnosed with Harlequin sign associated with Horner syndrome secondary to a mass effect with T1 and T2 nerve impingement. The normal reflexes and light-reactive pupils on physical examination help rule out other dysautonomic conditions such as Adie and Ross syndrome. Following the diagnosis, the patient continued radiation treatment for her underlying malignancy, leading to subsequent improvement in her facial flushing.

## Discussion

Harlequin sign is a rare but noteworthy clinical manifestation in which damage to sudomotor and vasomotor innervation from cervical sympathetic ganglia causes hemifacial paleness and anhidrosis on the affected side. The contralateral side subsequently reacts with compensatory thermoregulatory vasodilation and hypersudation.^1^^,^^2^ This results in a striking appearance characterized by a sharp midline dividing the pale and dry half from the flushed and moist half of the face. This cutaneous presentation, described by Lance and Drummond in 1988, was named after the red-and-black mask adorned by Harlequin, a 16th-century character from Italian *Commedia dell’Arte*.^1^ The terms “Harlequin sign” and “Harlequin syndrome” are often used interchangeably but should not be confused with Harlequin-type ichthyosis, a severe genetic condition that results in thickened plates of skin across the entire body of affected infants.

Harlequin sign is often associated with other dysautonomic syndromes, such as Horner syndrome, where disruption to cervical oculomotor neurons presents as the classic triad of Horner syndrome: partial ptosis of the upper eyelid, constriction of the pupil, and anhidrosis on the affected side.^3^ Oculosympathetic neurons exiting from T1 and sudomotor and vasomotor neurons from T2 and T3 ascend the sympathetic chain and synapse at the superior cervical ganglion,^3^^,^^4^ which is located at C3 to C4 near the ensheathed internal carotid artery and internal jugular vein. Lesions along the sympathetic pathway from the stellate ganglion and superior cervical ganglion can cause concurrent Harlequin sign and Horner syndrome. Disruptions extending below the T1 level are likely to cause Harlequin sign without Horner syndrome and may result in flushing and perspiration of the neck, chest, or upper limbs.^5^

In cases such as ours, the neuronal disruption is due to a secondary cause, which may include a malignancy in the cervical region (ie, Pancoast tumor), trauma, lesions of the subclavian artery and its branches, mediastinal lymphadenopathy, or temporal arteritis.^3^^,^^5^ Iatrogenic causes include recent surgical or anesthetic procedures, such as resection of a cervical or thoracic mass, internal jugular vein catheterization, or thoracic epidural analgesia.^5^ Secondary causes must be investigated before considering it to be idiopathic. The condition may be congenital in up to 6% of cases and could appear with or without Horner syndrome.^6^ According to a literature review by Guilloton et al^7^ on 108 cases of Harlequin sign, 59 cases (54.6%) were idiopathic and 49 cases (45.4%) were due to a secondary or iatrogenic cause.

Diagnosis of Harlequin sign concomitant with Horner syndrome relies on a detailed history encompassing past medical history, medications, medical treatments and interventions, family history, and known triggers of symptoms. A thorough neurologic examination is warranted and should entail measurement of pupillary diameter in darkness and light, inspection of upper eyelids for asymmetric ptosis, and evaluation of any other abnormal neurologic signs. The hemifacial flushing and hyperhidrosis are often exacerbated by heat exposure, exercise, emotional stress, or eating spicy foods; thus, a brief exercise test or heat exposure can be done to induce symptoms in the clinic.^8^ Depending on the anatomic location of the sympathetic neuronal lesion, the erythema and sweating can extend to the neck, arms, and chest.^5^ Cervical and thoracic magnetic resonance imaging scans can further elucidate the primary cause, which should be the focus for treatment and management. Once the cause is addressed, symptoms may subsequently resolve on their own, as in our patient. Most individuals with Harlequin sign do not require specific medical treatment as symptoms themselves are benign, so education and reassurance are the mainstays of management. However, botulinum toxin injections or contralateral sympathectomy may be considered to improve cosmesis if symptoms persist and affect quality of life.^9^

Harlequin sign is a remarkable clinical presentation reflecting a disruption of cervical sympathetic innervation that can be due to a wide range of etiologies, necessitating a detailed workup. Although our patient already had a known history of malignancy, such cutaneous signs may manifest as the first presenting sign of internal disease and should prompt physicians to assess for the underlying etiology and consider imaging to rule out malignancy.

## Conflicts of interest

None disclosed.
